# Cross organelle stress response disruption promotes gentamicin-induced proteotoxicity

**DOI:** 10.1038/s41419-020-2382-7

**Published:** 2020-04-03

**Authors:** Chinaemere Igwebuike, Julia Yaglom, Leah Huiting, Hui Feng, Joshua D. Campbell, Zhiyong Wang, Andrea Havasi, David Pimentel, Michael Y. Sherman, Steven C. Borkan

**Affiliations:** 1 Boston Medical Center, Department of Medicine, Renal Section, Boston, MA USA; 2 Boston University School of Medicine, Department of Biochemistry, Boston, MA USA; 3 Ariel University, Department of Molecular Biology, Ariel, West Bank, Israel; 4 Boston University School of Medicine, Department of Pharmacology and Experimental Therapeutics, Boston, MA USA; 5 Boston University School of Medicine, Department of Computational Biomedicine, Boston, MA USA; 6 Boston University School of Medicine, Department of Cardiology, Boston, MA USA

**Keywords:** Stress signalling, Experimental models of disease

## Abstract

Gentamicin is a nephrotoxic antibiotic that causes acute kidney injury (AKI) primarily by targeting the proximal tubule epithelial cell. The development of an effective therapy for gentamicin-induced renal cell injury is limited by incomplete mechanistic insight. To address this challenge, we propose that RNAi signal pathway screening could identify a unifying mechanism of gentamicin-induced cell injury and suggest a therapeutic strategy to ameliorate it. Computational analysis of RNAi signal screens in gentamicin-exposed human proximal tubule cells suggested the cross-organelle stress response (CORE), the unfolded protein response (UPR), and cell chaperones as key targets of gentamicin-induced injury. To test this hypothesis, we assessed the effect of gentamicin on the CORE, UPR, and cell chaperone function, and tested the therapeutic efficacy of enhancing cell chaperone content. Early gentamicin exposure disrupted the CORE, evidenced by a rise in the ATP:ADP ratio, mitochondrial-specific H_2_O_2_ accumulation, Drp-1-mediated mitochondrial fragmentation, and endoplasmic reticulum–mitochondrial dissociation. CORE disruption preceded measurable increases in whole-cell oxidative stress, misfolded protein content, transcriptional UPR activation, and its untoward downstream effects: CHOP expression, PARP cleavage, and cell death. Geranylgeranylacetone, a therapeutic that increases cell chaperone content, prevented mitochondrial H_2_O_2_ accumulation, preserved the CORE, reduced the burden of misfolded proteins and CHOP expression, and significantly improved survival in gentamicin-exposed cells. We identify CORE disruption as an early and remediable cause of gentamicin proteotoxicity that precedes downstream UPR activation and cell death. Preserving the CORE significantly improves renal cell survival likely by reducing organelle-specific proteotoxicity during gentamicin exposure.

## Introduction

Aminoglycoside-induced nephrotoxicity accounts for more than a quarter of clinical acute kidney injury (AKI) and often prompts drug discontinuation^1^. Gentamicin, the most commonly used aminoglycoside, primarily damages the proximal tubule epithelial cell due to luminal megalin/cubulin receptor-mediated drug accumulation^2^, and preventing gentamicin uptake by proximal tubule cells limits nephrotoxicity^3^. In prokaryotic cells, gentamicin suppresses protein synthesis by irreversibly binding the bacterial 30S ribosomal subunit^4^. Although their ribosomes differ, gentamicin also causes endoplasmic reticulum (ER) stress, activates the unfolded protein response (UPR), and damages mitochondria in eukaryotic cells^5–7^. Despite extensive investigation, a unifying mechanism of gentamicin-induced renal cell injury that incorporates mitochondrial injury, ER stress, and proteotoxicity is lacking and no effective therapy exists.

The observation that gentamicin targets the ER and mitochondria, coupled with the recent report that gentamicin causes characteristic errors in protein synthesis via binding of the eukaryotic 80S ribosome^8^, suggests that gentamicin-induced proteotoxicity causes renal cell injury. Proteotoxicity results from an imbalance between the burden of misfolded proteins due to translational errors and oxidative stress that exceed the ability of chaperones for protein refolding, repair, and degradation^9^. This concept of proteotoxicity potentially reconciles observations that gentamicin induces lethal endoplasmic reticulum stress, a primary regulator of the unfolded protein response (UPR)^10,11^. However, a biologic link between ER stress, mitochondrial injury, and cell death has been elusive.

Recently, histologic and functional connections link the mitochondria and ER through an interaction identified as the cross-organelle stress response (CORE). The CORE maintains normal protein conformation by coordinating energy consumptive protein folding with metabolism and the demand for cell chaperone-mediated folding^12^. The CORE physically and functionally resides at mitochondrial-associated membranes (MAMs) located between the cytosol, ER, and mitochondria that contain the physical sensors and effectors of these processes. Integrated crosstalk between the cytosol, ER, and mitochondria limit proteotoxicity caused by normal protein folding and/or downstream protein misfolding^12–14^. Excess oxidative stress or loss of cell chaperone activity disrupt the CORE^9,12^, causing dynamin-related protein-1 (Drp-1)-mediated mitochondrial fragmentation, ER–mitochondrial dissociation^12,15^, activation of pro-apoptotic CHOP^16^, and ultimately, cell death. When severe or prolonged, CORE disruption progresses to whole-cell perturbations of the unfolded protein response (UPR), a compensatory mechanism that initially attempts to restore proteostasis caused by mitochondrial and ER dysfunction induced by oxidative stress^16,17^. Although UPR activation often restores proteostasis, prolonged or severe UPR activation precipitates cell death via a BCL2 protein-dependent process^18,19^. Thus, CORE disruption and UPR activation potentially utilize interconnected cell death signal pathways.

ER stress has been implicated in causing ischemic and nephrotoxic acute kidney injury (AKI)^20^. The CORE is an attractive target for gentamicin-induced proteotoxicity because ER–mitochondrial collaboration prevents the toxic protein misfolding that accompanies normal protein synthesis^19^. To reconcile prior reports of gentamicin-induced cell injury with recent advances in the understanding of organelle crosstalk, we used an unbiased, high-throughput shRNA screening strategy to identify signal pathways disrupted by gentamicin in human proximal tubule cells, a primary target of nephrotoxic injury. The hypothesized pathways generated by this screen were validated through targeted testing of critical aspects of the CORE and UPR as well as the cellular response to a therapeutic intervention predicted to modify these pathways. Based on our screening data, we tested the hypothesis that early CORE disruption is a major mechanism of gentamicin-induced proteotoxicity and a therapeutic target.

This study describes CORE disruption as both an early marker and a novel therapeutic target during gentamicin-induced proximal tubule cell injury. Specifically, gentamicin increases mitochondrial-specific oxidative stress and rapidly disrupts CORE, resulting in marked mitochondrial fragmentation and ER–mitochondrion dissociation that predispose to untoward UPR activation and proteotoxic cell death. Conversely, reducing mitochondrial-specific oxidative stress preserves the CORE, reduces downstream UPR activation, and provides cytoprotection against gentamicin-induced proteotoxicity. For the first time, we suggest that early gentamicin-induced CORE disruption contributes to proteotoxicity and that CORE preservation limits toxic UPR activation and improves renal cell survival.

## Materials and methods

### Cell culture

Human kidney proximal tubule epithelial (HK-2) cells (ATCC CRL-2190) were cultured in Dulbecco’s Modified Eagles Medium (DMEM; Mediatech, Manassas, VA; 4.5 gm/L of glucose, 584 mg/L L-glutamine, without pyruvate) supplemented with 1% penicillin/streptomycin (Mediatech) and 10% fetal bovine serum (Equitech-Bio, Kerrville, TX) at 37 °C in 5% CO_2_ as previously described^21^.

### RNAi screening

An shRNA screening library with five distinct shRNAs directed against 5000 signaling genes (Cellecta, Mountain View, CA) was used to identify the biochemical events altered by gentamicin exposure as compared with vehicle-treated control. Human proximal tubule epithelial cells infected with the pooled lentiviral shRNA library were exposed to gentamicin at a dose sufficient to kill 80–90% of cells within 10 days, a model that resembles the time course of human acute kidney injury^22^. Vehicle (DMSO)-treated cells served as the experimental control in addition to a luciferase shRNA internal control. After harvesting chromosomal DNA, nested PCR was used to amplify 25,000 individually barcoded lentiviral shRNAs (five shRNA’s per gene) in vehicle control and gentamicin-treated cells. shRNA species were quantitatively identified by Ion Torrent sequencing of the unique barcode encoded in each lentivirus. Our custom-made software normalized individual shRNA abundance to five nonspecific luciferase shRNAs. If activation of a gene (i.e., signaling pathway component) either directly or indirectly promotes gentamicin toxicity, then shRNA-mediated inhibition of this signal will ameliorate toxicity and increase its relative abundance in surviving cells. In contrast, if a signal event promotes cell survival after gentamicin exposure, then inhibiting it with a specific shRNA increases cell toxicity and reduces its relative abundance in the surviving cells. Duplicate screens in gentamicin-exposed renal cells used a cutoff value of ±2.5 standard deviation change vs. vehicle control shRNA abundance.

### Pathway analysis

Gene sets identified in our screens were analyzed using the Ingenuity Pathways Analysis (IPA; Qiagen, Redwood City, CA) software package. In addition, a custom shRNA Analysis Program was developed. This pathway analysis facilitated a logical grouping of individual pathways into a “best fit” mechanism and identified potential drugs for preventing gentamicin toxicity^23,24^. “Best fit” was defined as the pathway that accounts for the majority of detected shRNA abundance changes in gentamicin-exposed human proximal tubule epithelial cells.

### ATP:ADP live cell assay

The ATP:ADP ratio was measured in live cells transfected with a fluorescent ATP:ADP reporter probe (Perceval, Addgene, Watertown, MA) using an Olympus DSU spinning disc microscope, as previously described^25^. The ratio of ATP to ADP was calculated using the ratio of 490 nm to 405 nm excitation and emission was collected through a 529/39-nm band-pass filter, and excitation and emission light was separated with a 490 nm short pass dichroic. An increase in ATP:ADP ratio presents as an increase in 490 nm, and a concurrent decrease in emission at 405-nm excitation. In contrast, a decrease in ATP:ADP ratio decreases emission at 490-nm excitation and also decreases the ratio.

### Mitochondrial-specific oxidative stress

MitoHyPer (Evrogen, Moscow, Russia, Cat #FP942), a variant of yellow fluorescent protein, was used to measure mitochondrial-specific hydrogen peroxide, as previously described^26^. This probe selectively measures namomolar amounts of H_2_O_2_ using the ratio of emission signals at 500 and 516 nm to differentiate bound vs. unbound H_2_O_2_. HyPer probe fused to dual mitochondrial targeting sequences derived from cytochrome c oxidase subunit VIII was expressed in cells using adenovirus under the control of the CMV promoter. Cells were imaged in wide field mode at 37 °C and 5% CO_2_ for 96 h following transfection using an inverted Olympus Spinning Disk confocal microscope. Spectrophotometric data were analyzed with NIS Elements software (Nikon) corrected for background GFP emission divided by background-subtracted emission. To avoid photobleaching, images were obtained at 5 min intervals to confirm stability and for 35 min after gentamicin and/or GGA addition.

### Mitochondrial morphology and quantification

Mitochondria were stained with MitoTracker (MitoTracker Red CMXRos #M7512 and MitoTracker Green FM #M7514). Mitochondrial interconnectivity, elongation, and morphology were measured using an Image J macro designed by Ruben K. Dagda (University of Pittsburg). The average circularity and perimeter/area ratio were used to measure mitochondrial length. Average circularity detected normal, elongated (less circular) vs. fragmented (more circular) mitochondria. Area to perimeter normalized to circularity was used to measure normal, interconnected mitochondria, and accounted for potential organelle swelling^27^.

### ER–mitochondrial co-localization

Mitochondria were stained with MitoTracker (MitoTracker Green FM #M7514), and ER were labeled with ER Tracker (ER-Tracker Red; BODIPY TR Glibenclamide). Co-localization was measured on Image J using Mander’s split correlation and the Pearson Correlation coefficient. Co-localization was proportional to fluorescence signal intensity, and was expressed in fraction of intensity values ranging from 0 to 1^28^. Real-time video imaging was obtained using a Nikon N-SIM super-resolution microscope.

### Chaperone function

Cell chaperone function was measured by microscale thermophoresis (Tycho^TM^, NanoTemper, Munich, Germany). This technique was performed in cell lysates according to the manufacturer’s instruction and measures total cell protein flexibility at increasing temperatures between 0 and 120 °C, an estimate of cell chaperone activity^29,30^. To quantify these results, the protein folding change ratio at 0 vs. 120 °C was calculated to express overall protein flexibility, a measure of chaperone function, in response to thermal stimulation.

### Luciferase enzyme activity assay

Total luciferase activity was measured in cells using an ATP luminescence kit (Life Technologies, Eugene, OR catalog #A22066) modified to reflect luciferase activity, in which substrate was not rate limiting. Light emission reflecting total luciferase activity was determined according to the manufacturer’s instructions in 12 identical samples in a 96-well plate, and the results were then averaged.

### Whole-cell oxidative stress assay

Live cell reactive oxygen species (ROS) were measured using a commercially available fluorogenic probe (Abcam Cellular ROS Assay Kit AB186027). The CellROX probe is localized to the cytoplasm and exhibits strong fluorogenic signal during whole-cell oxidation, especially oxidation by superoxide and hydroxyl radicals^31^. Cells were incubated in 5 μM of CellROX reagent for 30 min at 37 °C in accordance with the manufacturer’s instructions prior to live cell imaging.

### Beta amyloid and protein aggregate staining

Thioflavin T staining was used as a marker of ER stress and intracellular misfolded protein aggregation^32,33^. The fraction of cells containing thioflavin puncta was quantified using the Image J Particle Analysis. Number of puncta was normalized to cell nuclei stained with Hoechst 33342 (Cayman Chemical Cat No. 875756-97-1; 1 µg/mL). Thioflavin T puncta was measured by automated image cytometry via the Celigo Image Cytometer (Nexcelom; Lawrence, MA).

### Immunoblot analysis

Western blot analysis was performed using renal cell lysates as previously described^21^. Cell protein levels were measured using the BCA assay (Pierce), and equal amounts of protein (4–20 µg) were separated on 4–12% bis-tris polyacrylamide gels. Separated proteins were transferred to nitrocellulose membranes, and antigens were detected using specific primary antibodies (detailed below). MG132, a proteasome inhibitor, was added (10 µM for 4 h) prior to performing polyubiquitin immunoblots of cell lysates. Polyubiquitin blots were transferred overnight at 0.4 A using a previously described protocol^34^.

### Antibodies

UPR activation was measured using BiP (Cell Signaling C50B12), ATF6 (Abcam ab37149), XBP1 (Santa Cruz SC-7160), and CHOP (Cell Signaling L63F7) antibodies. Mitochondrial fission mediated by the ER was measured using the active (p-Ser616) and total forms of the mitochondrial pro-fission protein Drp-1 (Cell Signaling 3455 and Abcam #ab123599, respectively). Apoptosis was quantified using cleaved PARP (Cell Signaling #9541S) and the anti-apoptotic protein, Bcl-xL (Cell Signaling #2764S). Protein degradation was measured using a polyubiquitin antibody (Enzo Life Sciences BML-PW8810-0100). Oxidative stress was measured using 4-hydroxy-2-nonenal (Abcam ab46545) antibody. Equivalent sample loading was assessed using a beta-actin antibody (ThermoFisher #MA5-15739).

### Densitometry

After digitizing each immunoblot image (Hewlett-Packard, Desk Scan II), selected band densities were quantified using NIH Image J Software. Data are expressed as mean ± SE.

### Hsp70 induction

To induce Hsp70, cells were exposed to 50 μM geranylgeranylacetone (GGA) for 24 h prior to experiments. GGA caused a dose-dependent induction of Hsp70 (Supplementary Fig. 5A, B) that persisted for at least 96 h post-GGA exposure (Supplementary Fig. 5C, D).

### Cell survival

Survival was measured using a live/dead cell assay and automated image cytometry via the Celigo Image Cytometer (Nexcelom; Lawrence, MA). The live/dead cell assay used staining with Hoechst 33342 (Cayman Chemical Cat No. 875756-97-1; 1 µg/mL) and propidium iodide (ThermoFisher Cat No. P3566; 1.5 µM) for cell nuclei and membrane integrity, respectively. Stain intensity, cell size, and count measured in gentamicin-exposed cells were normalized to a vehicle-only control.

### Statistical analysis

Data were analyzed using Excel (Microsoft, Redmond, WA) or SigmaPlot software (Systat Software, Inc., San Jose, CA). Directional differences in relative immunoblot density and mitochondrial fragmentation were measured by one-tailed ANOVA. Statistical analysis was otherwise performed using a two-tailed, unpaired Student’s *t* test incorporating Bonferroni’s correction for more than two comparisons. Significance was determined as *P* < 0.05.

## Results

### shRNA screen identifies the cross-organelle stress response (CORE) as a mechanism of gentamicin-induced injury

**Fig. 1:**
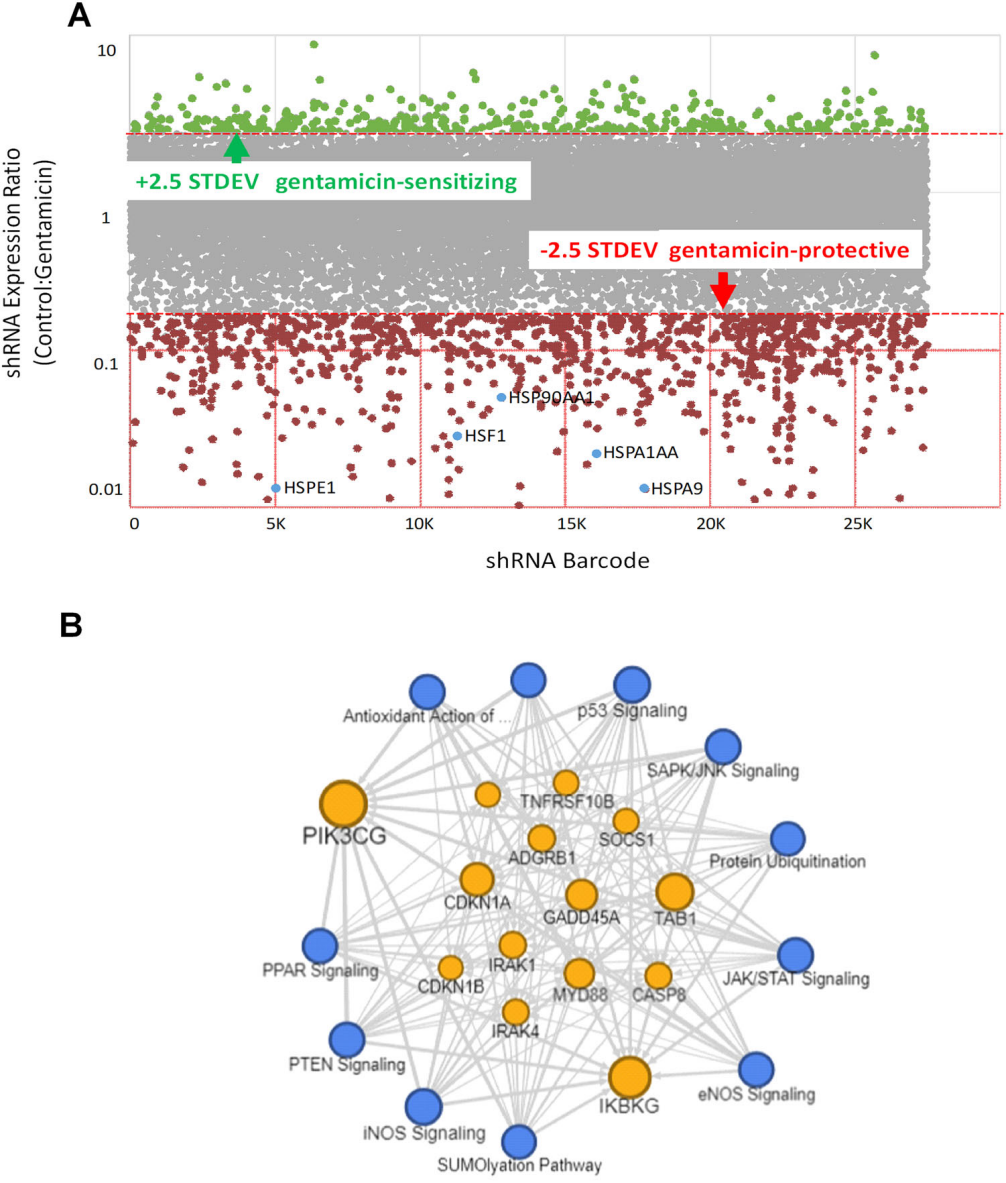
Pathway analysis of gentamicin-exposed human proximal tubule epithelial (HK-2) cells identified signal events in the Unfolded Protein Response (UPR) as potential contributors to injury. **a** HK-2 cells infected with an shRNA-based lentiviral signal gene library were exposed to gentamicin prior to RNAi analysis, and the scatterplot of shRNA abundance is shown. A threshold of ±2.5 STDEV was used to select significant changes in the abundance of signal-specific shRNA that either protected or sensitized renal cells to gentamicin. Red = increased abundance; Green = decreased abundance; Gray = unchanged *vs*. control shRNA. **b** Pathway analysis of significant shRNA changes yielded 226 genes within 11 distinct signal pathways that potentially mediate gentamicin-induced cell injury, including the UPR (shown in Supplementary Table 1). Blue nodes represent major signal pathways identified in our screen and orange nodes represent shared signal genes.

To determine a targetable mechanism of gentamicin-induced proximal tubule cell injury, we used an unbiased shRNA screen directed against cell signal pathway genes. The relative shRNA abundance ratios for 25,000 shRNAs directed against 5000 signal genes in gentamicin-exposed human proximal tubule epithelial cells (HK-2) in a representative screen are shown in Fig. 1a. This screen yielded 226 signal genes that were either significantly over- or underrepresented in gentamicin-exposed cells relative to vehicle controls. Repeated screens revealed a strong correlation and high degree of experimental reproducibility between shRNA abundance observed in independent experiments (*r*^2^ = 0.7725; Supplementary Fig. 1).

Pathway analysis of the shRNA screen identified 11 signal pathways with functions critical to the UPR (Table 1). Furthermore, the abundance of key UPR signal RNAs were decreased by gentamicin exposure, suggesting that UPR signals are cytoprotective. Analysis also revealed prominent protein degradation mechanisms including ER oxidant stress pathways including iNOS signaling (9/35 genes), the protein ubiquitination pathway (38/172 genes), and mitochondria-mediated cell death pathways (27/396 genes). In addition, mitochondrial biogenesis and mTOR have been directly linked to proteotoxicity and the UPR^35,36^. Taken together, these analyses suggest that the CORE and UPR mediate gentamicin-induced proximal tubule cell injury (Fig. 1b). Among individual genes in gentamicin-exposed cells, we also detected a significant reduction in the abundance of several cell chaperones likely involved in preventing proteotoxicity, including heat shock factor-1 (HSF-1), the primary regulator of Hsp70, and Hsp70 itself (Supplementary Table 1 and Supplementary Fig. 1C). Using this mechanistic insight to link CORE disruption with altered proteostasis, we hypothesized that geranylgeranylacetone (GGA), an HSF-1 inducer, and a robust promoter of both Hsp70 expression^37^ and regulation of the CORE^12^, might ameliorate gentamicin-induced proteotoxicity.

**Table 1:** Effect of gentamicin exposure on potential proteotoxic pathways in human proximal tubule epithelial cells.

Key signal pathways	Pathway effects	Fraction pathway representation	*Z*-score	*P*-value	Citations
EIF2	Unfolded protein response/ER stress	28/208 (0.139)	−3.301	4.47E-14	^76^
PTEN	mTOR/autophagy/cell survival	9/119 (0.076)	+2.995	3.77E-04	^77,78^
SAPK/JNK	Cell survival/Bax activation	13/98 (0.122)	−2.309	5.45E-06	^79,80,81^
p53	Oxidative stress/apoptosis	14/111 (0.126)	+1.265	6.28E-07	^82,83^
eNOS	Mitochondrial biogenesis	10/43 (0.227)	+0.707	9.18E-08	^84^
iNOS	Oxidative stress/apoptosis	9/161 (0.056)	−1.890	3.16E-03	^85,86^
Antioxidant Action (Vit C)	Limits ROS injury/mitochondrial biogenesis	11/92 (0.109)	+1.897	9.25E-05	^87,88^
Sumoylation	Unfolded protein response/ER stress	11/102 (0.088)	+1.667	9.70E-04	^89,90^
PPAR	Mitochondrial biogenesis	7/96 (0.062)	−2.001	1.53E-02	^91,92^

Eighty-two percent (9 of 11) pathways that were significantly altered by gentamicin exposure in the shRNA screens (Fig. 1b) are either directly related to the unfolded protein response (UPR) or to key UPR components including ER stress, mitochondrial injury, and/or apoptotic cell death (see “Discussion”).

### CORE dysfunction associated with impaired mitochondrial function and dynamics during gentamicin-induced injury

**Fig. 2:**
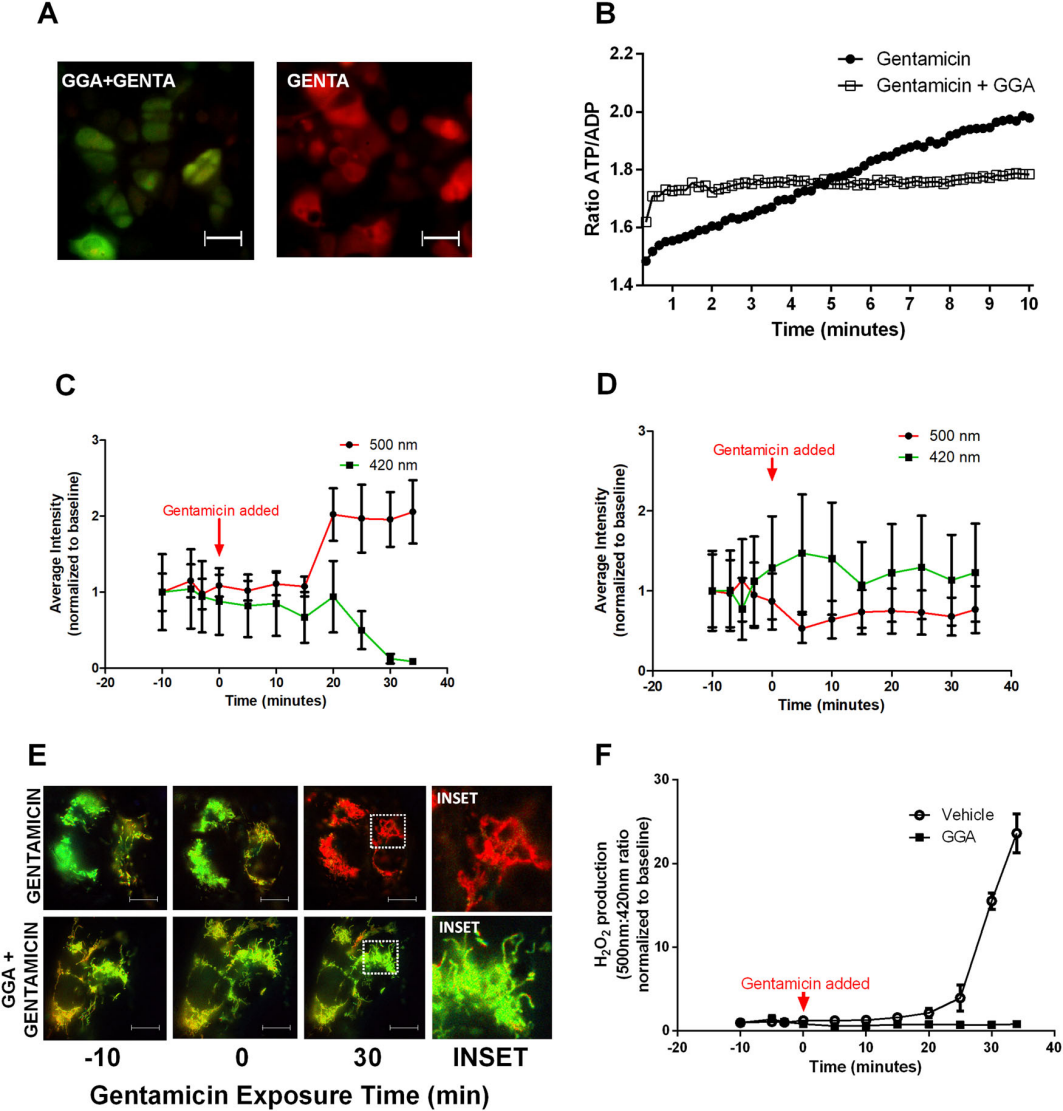
Gentamicin exposure increases mitochondrial ROS and the ATP:ADP ratio in HK-2 cells within 30 min and GGA ameliorates gentamicin-induced mitochondrial ROS and preserves the ATP:ADP ratio. **a** Fluorescent images of the ATP:ADP ratio measured by a probe sensitive to both ATP and ADP show that gentamicin exposure triggers a larger proportion of ATP (red) relative to ADP (green) in renal cells. **b** Serial ATP:ADP ratio measurements show that gentamicin causes a gradual increase in ATP:ADP ratio, whereas GGA preserves the baseline ATP:ADP ratio in gentamicin-exposed cells. Data are normalized to control. **c** Cells exposed to gentamicin show an increase in mitochondrial H_2_O_2_ measured by the mitochondrial HyPer probe as a decrease in the excitation peak at 420 nm proportional to the increase in the peak at 500 nm. **d** GGA treatment prior to gentamicin exposure ameliorated this increase in mitochondrial H_2_O_2_. **e** Ratiometric images of 500 nm:420 nm excitation in cells with MitoHyPer probe show a similar increase in mitochondrial ROS (denoted in red) in vehicle control cells relative to GGA-treated cells. **f** Vehicle-treated cells exhibited a marked increase in mitochondrial H_2_O_2_ during gentamicin exposure and GGA treatment completely prevented mitochondrial H_2_O_2_ accumulation during gentamicin exposure. *n* = 4, Error bars = SEM. Bars = 5 µm.

The CORE regulates proteostasis partly by facilitating ATP transfer from the mitochondria to the endoplasmic reticulum to support energetically unfavorable protein refolding^38^. This phenomenon is observed within proximal tubule cells, where live cells increased ATP, decreased ADP, and increased the ATP:ADP ratio during gentamicin exposure (Fig. 2a). This suggests that early ER stress initially manifests as increased energy demand. In contrast, the addition of geranylgeranylacetone (GGA) largely prevented these changes in ATP and ADP, and prevented the increased ATP:ADP ratio caused by gentamicin (Fig. 2b). The mitochondrion is a major source of intracellular ROS and CORE disruption increases mitochondrial ROS production^39^. To assess organelle-specific ROS, a mitochondrial-targeted hydrogen peroxide probe was introduced. MitoHyper detected organelle-specific oxidant accumulation in human proximal tubule cells within 30 min of gentamicin exposure (Fig. 2c). In contrast, GGA treatment eliminated mitochondrial ROS accumulation in gentamicin-exposed cells (Fig. 2d, e, f). A time-dependent measurement of luciferase activity, a marker of chaperone function^40,41^ that reflects cell enzyme activity, showed a corresponding decrease within 5 h of gentamicin exposure, and achieved significance after 48 h of drug exposure (*P* < 0.05; Supplementary Fig. 2A, B).

**Fig. 3:**
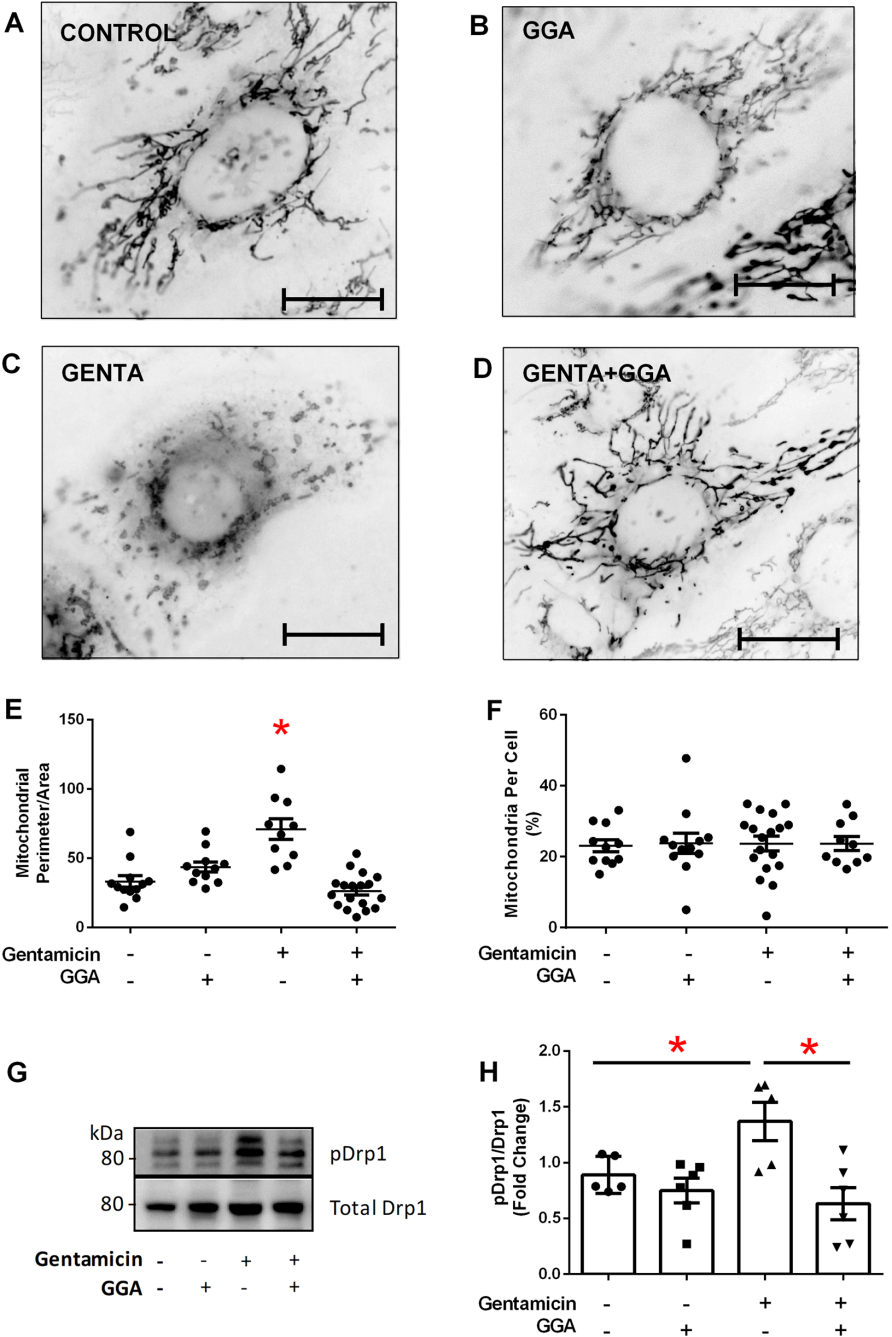
Gentamicin exposure caused mitochondrial fragmentation in human renal cells. Cells stained with MitoTracker Red were converted to greyscale and color inverted to improve mitochondrial visualization. **a** Untreated control with healthy mitochondria. **b** GGA exposure alone did not significantly alter mitochondrial morphology. **c** Gentamicin (GENTA) caused significant mitochondrial fragmentation. **d** GGA (GENTA + GGA) inhibited gentamicin-induced mitochondrial fragmentation; bar = 5 µm. **e** Gentamicin caused significant fragmentation evidenced by an increased perimeter:area ratio of mitochondria per cell; GGA prevented this change; *n* = 12–18. **f** Protection against gentamicin-induced mitochondrial fragmentation by GGA was independent of de novo mitochondrial biogenesis as measured by percentage of cell area containing mitochondria; *n* = 12–18. **g** Gentamicin-induced mitochondrial fragmentation correlated with an increase in both the steady-state level of pro-fission p-ser637 Drp-1 as well as the p-Drp-1:total Drp-1 ratio. **h** GGA significantly reduced the p-ser637 Drp-1:total Drp-1 ratio in gentamicin-exposed cells; *P < 0.05; *n* = 6.

To assess CORE disruption in gentamicin-induced proximal tubule injury, mitochondrial metabolism, dynamics, and inter-organelle crosstalk were examined. Compared with elongated, filamentous control mitochondria (Fig. 3a), organelle swelling and fragmentation occurred within 60 min of gentamicin exposure (Fig. 3c). In contrast, GGA treatment alone preserved healthy mitochondrial morphology (Fig. 3b) and prevented fragmentation in gentamicin-exposed cells (Fig. 3d). Morphologic quantification showed that GGA significantly prevented gentamicin-induced swelling and fragmentation (*P* < 0.05, Fig. 3e). The protective effect of GGA on mitochondrial morphology was independent of de novo organelle biogenesis (Fig. 3f). Since CORE-mediated mitochondrial fragmentation is regulated by localized dynamin-related protein-1 (Drp-1) activation on the mitochondrial-associated membrane (MAM)^13^, we compared the ratio of activated Drp-1 to total Drp-1 in gentamicin-exposed cells^18,42^. Gentamicin exposure significantly increased the ratio of p-Drp-1/total Drp-1 (Fig. 3g, h), whereas GGA significantly decreased this ratio to baseline levels, despite gentamicin exposure. These observations show that GGA prevents the mitochondria-specific oxidative stress, Drp-1 activation, and mitochondrial fragmentation caused by gentamicin.

**Fig. 4:**
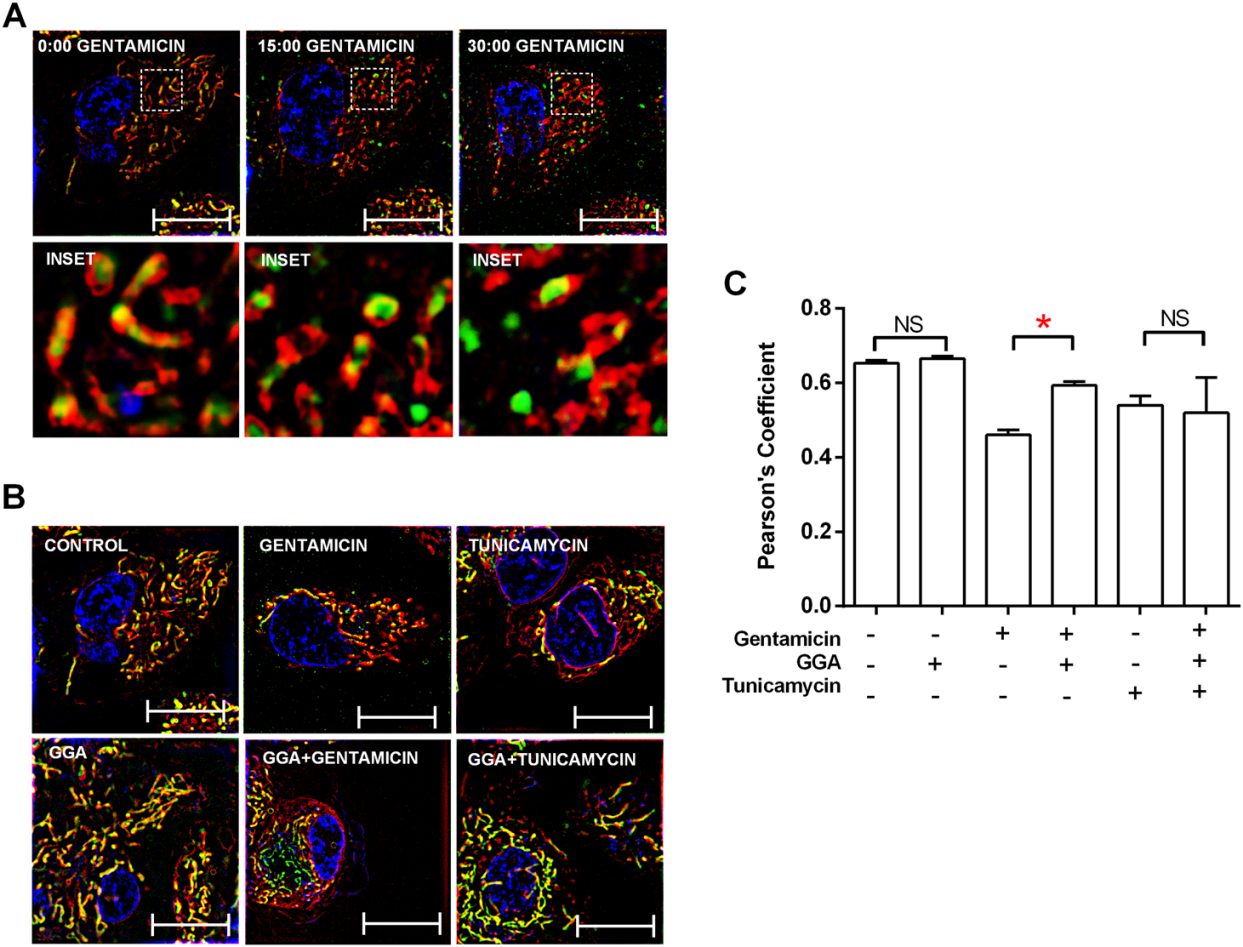
Gentamicin exposure causes mitochondrial–ER dissociation. Representative micrographs show mitochondria, endoplasmic reticulum (ER) and nuclei stained with MitoTracker Green, ER-Tracker Red, and Hoechst dye (blue), respectively. **a** At baseline, cells contain elongated mitochondria that co-localize with ER. After 15 min gentamicin exposure, mitochondria appear severely fragmented although ER and mitochondria remain co-localized. After 30 min of gentamicin exposure, fragmented mitochondria dissociate from ER. Bars = 5 µm. Insets contain magnified images highlighting mitochondrial–ER proximity. **b** Compared with control (left upper panel), GGA alone did not alter baseline mitochondrial morphology (left lower panel), but prevented both mitochondrial fragmentation and dissociation from ER in gentamicin-exposed cells *(*lower center panel vs. upper center panel*)*. Tunicamycin also caused mitochondrial fragmentation followed by organelle dissociation (right upper panel). In tunicamycin-exposed cells, GGA reduced mitochondrial fragmentation but did not prevent mitochondrial–ER dissociation (right lower panel vs. right upper panel); Bars = 5 µm. **c** GGA significantly preserved mitochondrial–endoplasmic reticulum co-localization (Pearson’s Coefficient) during 30-min gentamicin exposure; *P < 0.05; *n* = 108–124 mitochondria.

MAMs coordinate the ER–mitochondrial association crucial to CORE and cell survival by limiting toxicity caused by the misfolded proteins that activate lethal UPR^43^. We hypothesized that gentamicin-induced CORE disruption alters MAMs and dissociates ER from mitochondria in human proximal tubule epithelial cells. To determine whether gentamicin exposure causes ER–mitochondrial dissociation, the intracellular co-localization of mitochondria and ER was quantified using Pearson’s coefficient of the respectively stained organelles. In healthy cells, mitochondriaand ER were closely associated (Fig. 4a). Within 30 min, gentamicin caused a marked mitochondrial–ER fragmentation followed. In healthy cells, mitochondria and ER were closely associated (Fig. 4a). Within 30 min, gentamicin caused a marked mitochondrial–ER fragmentation followed by dissociation (Fig. 4a, b, upper middle panel; Supplementary Fig. 3 video). In contrast, GGA treatment prevented gentamicin-induced mitochondrial–ER dissociation (Fig. 4b, lower middle panel), a protective effect that reached statistical significance (Fig. 4c). Similar to gentamicin, tunicamycin caused mitochondrial fragmentation and mitochondrial–ER dissociation (Fig. 4b, upper right panel). In these tunicamycin-exposed cells, GGA treatment prevented mitochondrial fragmentation and partially reduced mitochondrial–ER dissociation (Fig. 4b, lower right panel). Taken together, these data suggest that gentamicin rapidly causes metabolic and oxidative stress in mitochondria that promotes CORE disruption, characterized by mitochondrial fragmentation and mitochondrial–ER dissociation. Furthermore, GGA reverses these untoward effects of gentamicin on the CORE.

### Gentamicin-induced CORE dysfunction precedes whole-cell oxidative stress, protein degradation, and protein misfolding

**Fig. 5:**
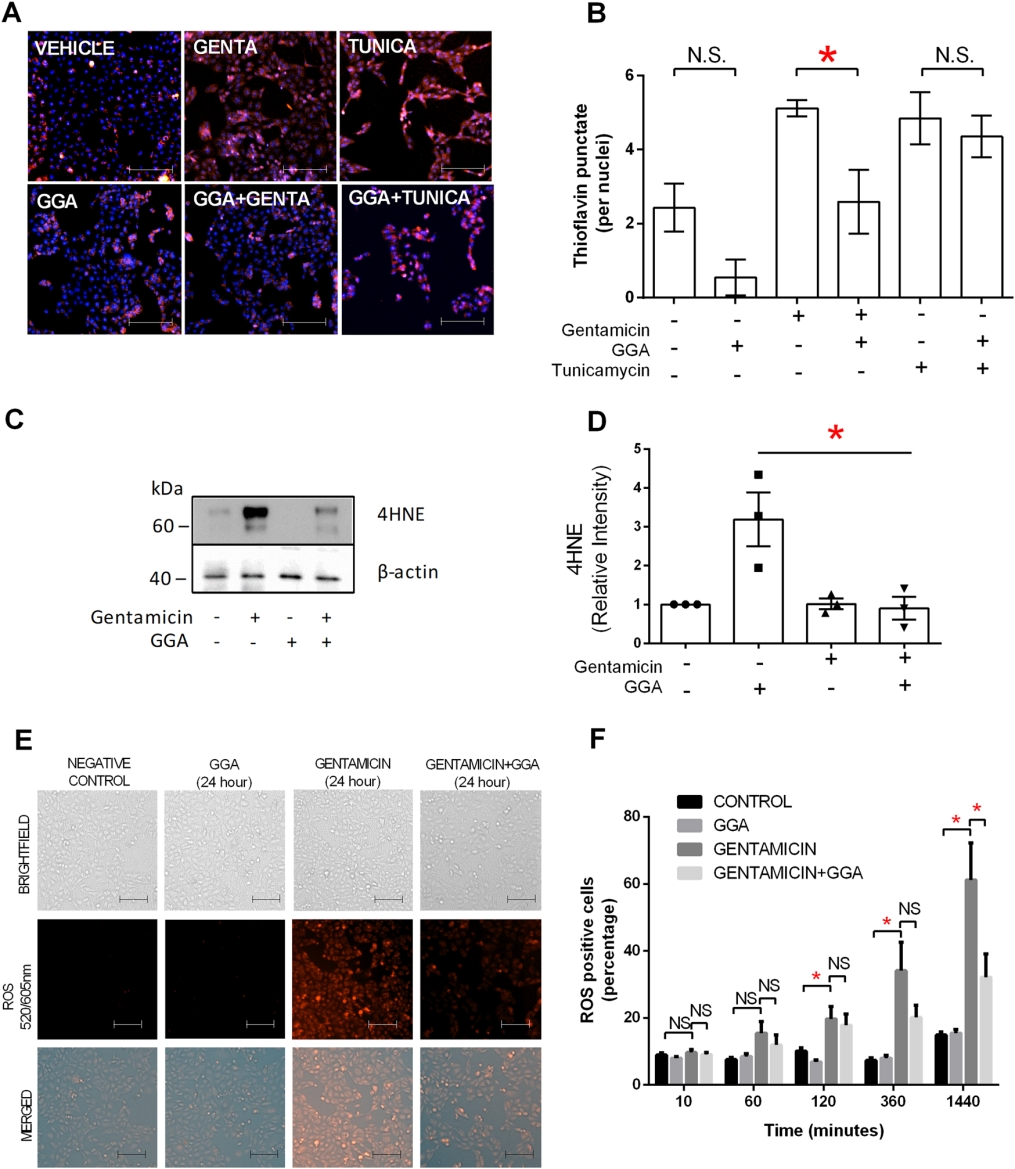
Effect of gentamicin or tunicamycin vs. GGA on whole-cell oxidative stress. **a** Misfolded protein load in gentamicin (GENTA), tunicamycin (TUNICA), and/or GGA-exposed cells assessed by Thioflavin T staining. Red = Thioflavin T; blue = Hoechst stained nuclei; bars = 50 µm. **b** Thioflavin T puncta quantified in gentamicin or tunicamycin vs. GGA-exposed renal cells. **c** Gentamicin increased oxidative stress evidenced by increased steady-state 4HNE content. **d** GGA significantly reduced 4HNE content in gentamicin-exposed cells. **e** Content of ROS in renal cells after variable duration exposure to gentamicin assessed by fluorescent whole-cell oxidative stress assay. **f** GGA significantly reduced whole-cell ROS content in gentamicin-exposed cells; bars = 50 µm; NS = nonsignificant; *P < 0.05.

**Fig. 6:**
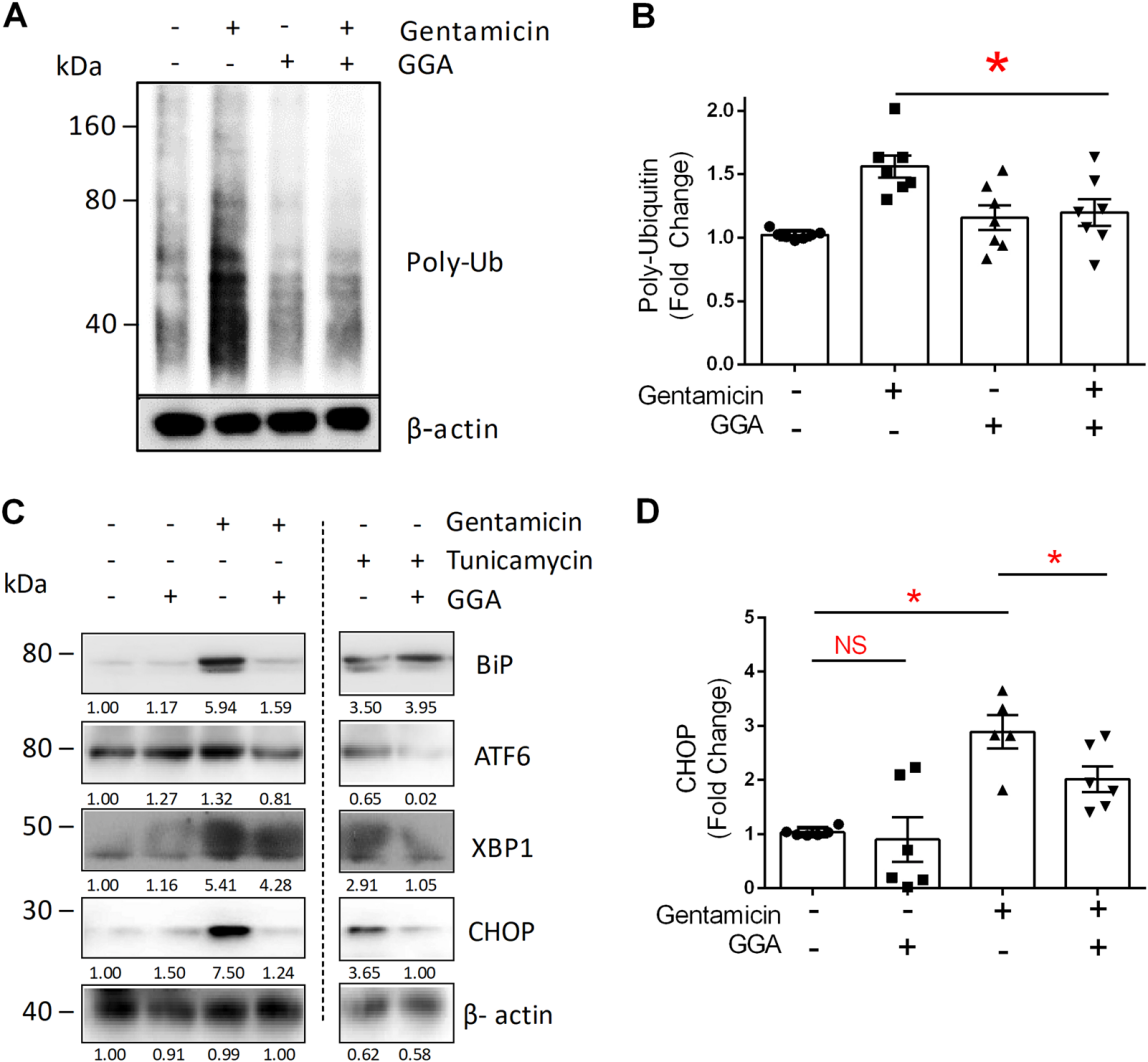
GGA reduces protein polyubiquitination and UPR activation in gentamicin-exposed renal cells. **a** Gentamicin increased protein degradation measured by polyubiquitination in cell lysates. **b** In gentamicin-exposed cells, GGA significantly reduced protein polyubiquitination to the baseline level; *n* = 7. **c** Immunoblot analysis shows BiP induction and downstream activation of all three UPR markers: ATF6, XBP1, and CHOP vs. beta-actin loading control. GGA decreased BiP, ATF6, and CHOP induction in gentamicin-exposed cells. Tunicamycin also increased steady-state BiP as well as the three UPR markers; GGA reduced all three UPR markers without preventing BiP induction; **d** GGA significantly reduced CHOP accumulation in gentamicin-exposed cells; Error bars = SEM; *P < 0.05; *n* = 6.

CORE disruption contributes to oxidative stress partly by generating free radicals during protein synthesis^44^. Despite mitochondrial ROS accumulation, no increase in whole-cell oxidative stress could be detected by early Thioflavin T puncta staining (Supplementary Fig. 3A), 4-hydroxynonenal (4HNE) immunoblot analysis (Supplementary Fig. 3B), or fluorescent measurements of whole-cell ROS accumulation (Supplementary Fig. 3C, D) during the first hour of gentamicin exposure. In contrast, 24-h gentamicin exposure increased oxidative stress, evidenced by increased Thioflavin T puncta staining (Fig. 5a, b), 4HNE accumulation (Fig. 5c, d), and caused a progressive increase in whole-cell oxidative stress between 2 and 24 h of gentamicin exposure (Fig. 5e, f). GGA significantly reduced Thioflavin T puncta staining (Fig. 5a, b), 4HNE content (Fig. 5c, d), and whole-cell oxidative stress (Fig. 5e, f). In contrast, GGA failed to reduce Thioflavin T puncta (Fig. 5a, b) or whole-cell oxidative stress (Fig. 5e, f) in tunicamycin-exposed cells, indicating that the mechanism of tunicamycin injury likely differs from that of gentamicin. Since the ubiquitin–proteasome system is highly responsive to the protein misfolding caused by oxidant stress^45^, the effect of gentamicin on protein polyubiquitination was measured as a surrogate for the ubiquitin–proteasome system^46^. Gentamicin exposure for 24 h significantly increased protein polyubiquitination, and GGA markedly reduced aberrant polyubiquitination (Fig. 6a, b). These results show that mitochondrial oxidative stress and CORE disruption precede a detectable rise in whole-cell oxidative stress and misfolded protein content.

### Gentamicin causes UPR activation and cell chaperone dysfunction

Misfolded protein accumulation is the primary stimulus for the UPR in healthy cells, but prolonged or severe proteotoxic stress overwhelms the refolding capacity of cell chaperones and causes maladaptive UPR^46,47^. Maladaptive UPR impairs the machinery responsible for protein degradation causing both ER dysfunction and cell death^46,47^. BiP (or GRP 78, a member of the HSP70 family) is an ER-specific, stress response chaperone, and master UPR regulator^48^. In healthy cells, BiP binds and suppresses three distinct transmembrane UPR transcriptional factors, including: inositol-requiring enzyme 1α (IRE1α), protein kinase RNA-like ER kinase (PERK), and activating transcription factor 6 (ATF6)^49^. During ER stress, however, BiP preferentially binds misfolded proteins, activating all three transcriptional elements of the UPR. In renal cells, gentamicin exposure increased the steady-state content of BiP (Fig. 6c, upper panel). Despite increased BiP, gentamicin markedly activated ATF6, XBP1, and CHOP, UPR elements downstream of IRE1α and PERK^50,51^, respectively (Fig. 6c). Similarly, tunicamycin, a positive control that inhibits glycoprotein synthesis^52^, modestly increased BiP content and also activated UPR (Fig. 6c). GGA treatment significantly reduced BiP, ATF6, and CHOP in gentamicin-exposed cells, and reduced ATF6, XBP1, as well as CHOP during tunicamycin exposure (Fig. 6c, d). These results suggest that gentamicin, similar to tunicamycin, activates UPR in human proximal tubule cells and that GGA reduces maladaptive UPR activation. A reduction in cell chaperone function would likely contribute to gentamicin-induced proteotoxicity. Gentamicin exposure caused a significant decrease in temperature-dependent protein flexibility, a measure of cell chaperone health (Supplementary Fig. 6A). In contrast, GGA partially restored protein flexibility (Supplementary Fig. 6B), consistent with the >fourfold increase in cell chaperone content induced by this agent (Supplementary Fig. 5B) that persisted for at least 96 h (Supplementary Fig. 5C, D).

### Preserving the CORE reduces cell death markers and improves cell survival

**Fig. 7:**
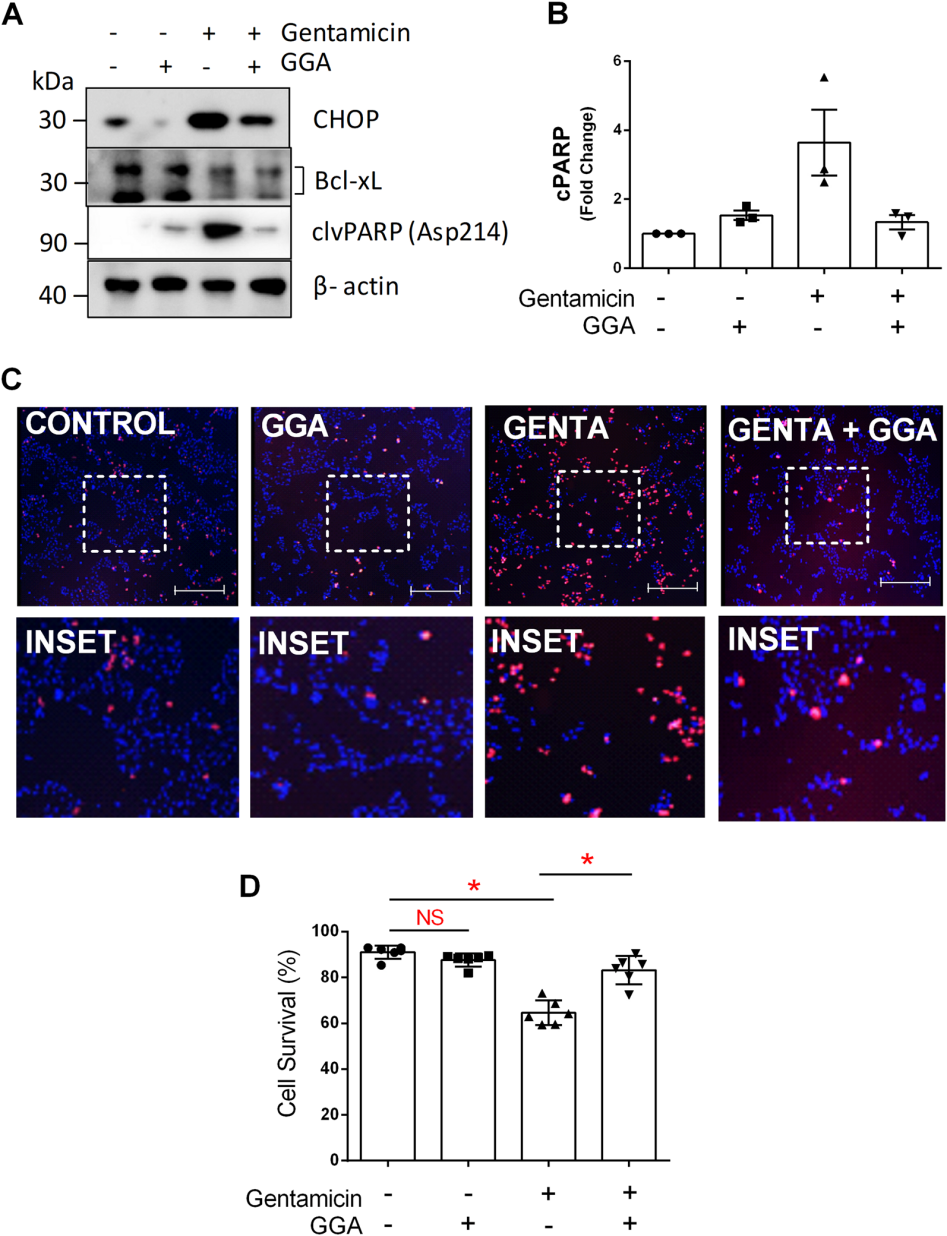
GGA reduced pro-apoptotic markers, and improved cell survival. **a** Gentamicin exposure increased apoptosis markers including cleaved PARP (clvPARP) and CHOP, and also decreased Bcl-xL content. **b** GGA significantly reduced cleaved PARP during gentamicin exposure. **c** Gentamicin increased the fraction of dead cells, and decreased total cell number as measured by propidium iodide and Hoechst staining, respectively (Genta vs. Control panels); bars = 50 µm. **d** Gentamicin significantly reduced cell viability, whereas GGA significantly improved survival; *P < 0.05; *n* = 6.

To identify the mechanism of gentamicin-induced UPR activation and cell death in proximal tubule cells, we measured the steady-state content of Bcl-xL, an anti-apoptotic BCL2 protein partially regulated by CHOP^20^, as well as PARP cleavage, a measure of apoptosis. Gentamicin exposure reduced Bcl-xL but increased CHOP content, a maladaptive marker of the UPR (Fig. 7a). Gentamicin also increased cleaved PARP (clvPARP) (Fig. 7a), an effect that achieved statistical significance (*P* < 0.05, Fig. 7b). These changes corresponded with decreased survival in gentamicin-exposed renal cells (Fig. 7c, d). In contrast, GGA reduced CHOP, significantly decreased cleaved PARP (Fig. 7a, b) and significantly improved survival in gentamicin-exposed cells (Fig. 7d), despite a similar fall in Bcl-xL content. Taken together, these data support the hypothesis that gentamicin causes cell death partly by increasing the accumulation of CHOP, the primary UPR death arm responsible for PARP cleavage, and Bax-mediated cell death^53^. In contrast, GGA inhibits gentamicin-induced CHOP expression and promotes renal cell survival.

## Discussion

CORE regulates ER–mitochondrial interactions, and was first identified in 2016 as an intrinsic mechanism for preventing proteotoxicity^12,54–56^. Mitochondrial-associated membranes (MAMs) mediate interactions between mitochondria and ER in proximal tubule cells, and the protein resorption requirements of the proximal tubule likely necessitate this inter-organelle relationship^43^. In healthy cells, the CORE limits proteotoxicity partly by providing additional mitochondrial ATP for protein refolding in the ER^38^. This is consistent with the initial rise in ATP:ADP ratio detected in human proximal tubule cells within minutes of gentamicin exposure. In a similar time frame, gentamicin causes mitochondrial-specific oxidative stress well before measurable changes in whole-cell oxidative stress or misfolded protein content are detected. These remarkable findings suggest that compartment-specific stress is an early harbinger of gentamicin-induced proteotoxicity that ultimately activates untoward UPR and cell death.

Compartment-specific oxidative stress causes mitochondrial dysfunction^57^ and mitochondrial fragmentation partly via its effects on Drp-1, a primary stress mediator and a key component of CORE-mediated mitochondrial fragmentation^58,59^. In gentamicin-exposed cells, we show that fragmentation precedes mitochondria–ER dissociation, likely reflecting the loss of mitochondrial-associated membranes (MAMs) responsible for organelle interaction^43^. Taken together, the early rise in the ATP:ADP ratio, mitochondrial-specific ROS accumulation, Drp-1-mediated mitochondrial fragmentation, and mitochondrial–ER dissociation provide clear evidence of CORE disruption before increases in either whole-cell oxidative stress or the misfolded protein load occurs. Although the signals that disrupt MAMs and CORE are disputed^60^, it is likely that organelle-specific stress is a primary trigger. This primary trigger of organelle-specific stress is further corroborated with recent descriptions of a novel form of localized mitochondrial UPR (UPR^mt^), which responds to excess ROS^59,61^.

Mitochondrial dynamics (i.e., the balance between organelle fission and fusion) play a major role in stimulating both local and whole-cell UPR responses^17^. The Drp-1-mediated mitochondrial shape change that occurs during early gentamicin exposure has recently been linked to UPR activation via altered mitochondrial calcium flux^62^, suggesting a causal link between mitochondrial fragmentation and the UPR activation observed in our studies. Specifically, Drp-1-mediated changes in mitochondrial morphology appear to be caused by the PERK arm of the UPR during endoplasmic reticulum stress^63^. Lipid and calcium perturbations, as well as Golgi-derived oxidative stress, have also been implicated in disrupting the CORE and causing both mitochondrial dysfunction and fragmentation^54,64^^65^, . Regardless of the trigger, CORE disruption compromises cell metabolism, mitochondrial–ER crosstalk, and contributes to lethal UPR activation by exacerbating proteotoxicity^12^.

This study shows that gentamicin exposure causes renal cell injury most likely by creating an imbalance between the burden of misfolded proteins and the chaperone/protein degradation machinery available to process them^13–18,23^. This imbalance is an important cause of proteotoxicity and contributes to organ failure during acute kidney injury^20,37^. In our shRNA screen, genes that alter survival in gentamicin-exposed human renal cells have been directly or indirectly linked to proteotoxicity and UPR. Specifically, EIF2, PTEN, mitochondrial biogenesis, and mTOR have been directly implicated in the UPR^35,36,63,66^, whereas oxidative stress has been linked to iNOS, intrinsic antioxidants, and P53^67^. In addition to these screening results and the evidence of reduced chaperone function in gentamicin-exposed cells, chaperones are an ideal therapeutic target due to their potential protective effects on both CORE and UPR pathways, and robust inducibility by GGA. Specifically, GGA induces Hsf-1, a key CORE regulator^12^, and upregulates Hsp70, a chaperone that reduces UPR activation by refolding cytosolic proteins, by promoting ER-linked misfolded protein degradation^48^ and perhaps by stabilizing the CORE^12^. We observed that GGA markedly induced Hsp70 expression and reduced the burden of misfolded proteins, protein polyubiquitination, and lethal UPR activation. Enhanced chaperone production may also have direct protective effects on mitochondrial morphology and ER–mitochondrial association. Additional mitochondrial chaperones such as Hsp60, Hsp10, or Grp75, a chaperone that resides at the ER–mitochondrial interface, may also regulate cytoprotection^68^.

UPR is situated to mediate both adaptive and maladaptive mechanisms that account for the diverse phenotype observed during gentamicin-induced proximal tubule cell death. UPR normally limits cell stress by increasing protein refolding and mitigating CHOP activation, a major contributor to cell death^69,70^. However, if cell stress is prolonged or irreversible, the balance between misfolded proteins and available chaperones is perturbed. In this untoward scenario, the UPR becomes a cell death pathway by activating CHOP, suppressing anti-apoptotic Bcl-xL^71,72^, and promoting the translocation of Bax from the cytosol to mitochondria^73^. In this study, CHOP accumulation was associated with decreased anti-apoptotic Bcl-xL content, PARP cleavage, and nuclear condensation. These findings suggest that Bax is likely to mediate cell death downstream of CHOP in gentamicin-exposed cells. In our pathways analysis, the presence of ER-stress markers, stress kinases SAPK/JNK, and altered protein SUMOylation known to regulate ubiquitination and protein degradation^74^ implicates maladaptive UPR activation during nephrotoxic stress.

In addition to preserving the CORE, GGA enhanced cell chaperone function, decreased protein misfolding and polyubiquitination, reduced ER stress (BiP content), and improved proximal tubule cell survival during gentamicin exposure. A reduction in the burden of toxic, misfolded proteins was accompanied by a decrease in the expression of maladaptive UPR markers including CHOP and cleaved PARP, and improved cell survival. Interestingly, gentamicin persistently activated XBP1 even in the presence of GGA. XBP1 is a downstream component of the IRE1α arm and upregulates UPR by stimulating the ER-stress element^75^. Our results with GGA suggest that XBP1 activation may be independent of Hsp70 or that other chaperones may be involved in regulating the IRE1α pathway. Induction of a more diverse array of protein chaperones identified in our shRNA screen, including select mitochondrial HSPs, might decrease XBP1 activation and afford more complete protection against gentamicin-induced cell injury.

In summary, proteotoxicity caused by early CORE disruption and untoward UPR activation is a unifying mechanism for gentamicin-induced proximal tubule cell death. This mechanism integrates compartment-specific oxidative stress during mitochondrial–ER crosstalk and provides an early marker of injury that occurs prior to changes in whole-cell protein misfolding or cell death. During gentamicin exposure, CORE disruption is a precursor to the proteotoxic events that overwhelm the cellular protein folding machinery, impair the ubiquitin–proteasome system, activate maladaptive UPR, and cause regulated cell death. Our findings suggest that gentamicin-induced mitochondrial ROS accumulation, Drp-1-mediated mitochondrial fragmentation, and ER–mitochondrial dissociation are the earliest pathological events described to date. Furthermore, the CORE may be a rationale target for limiting cell death caused by other proteotoxic insults.

## Supplementary information

Supplemental Figure 1

Supplemental Figure 2

Supplemental Figure 3

Supplemental Figure 4

Supplemental Figure 5

Supplemental Figure 6

Supplemental Table 1

Supplemental Figures Legends
