# Supplementary figures and images for: Cross organelle stress response disruption promotes gentamicin-induced proteotoxicity
Source: Cell Death Dis. 2020 Apr 3;11(4):217. doi: 10.1038/s41419-020-2382-7 (PMC7125232; doi:10.1038/s41419-020-2382-7)

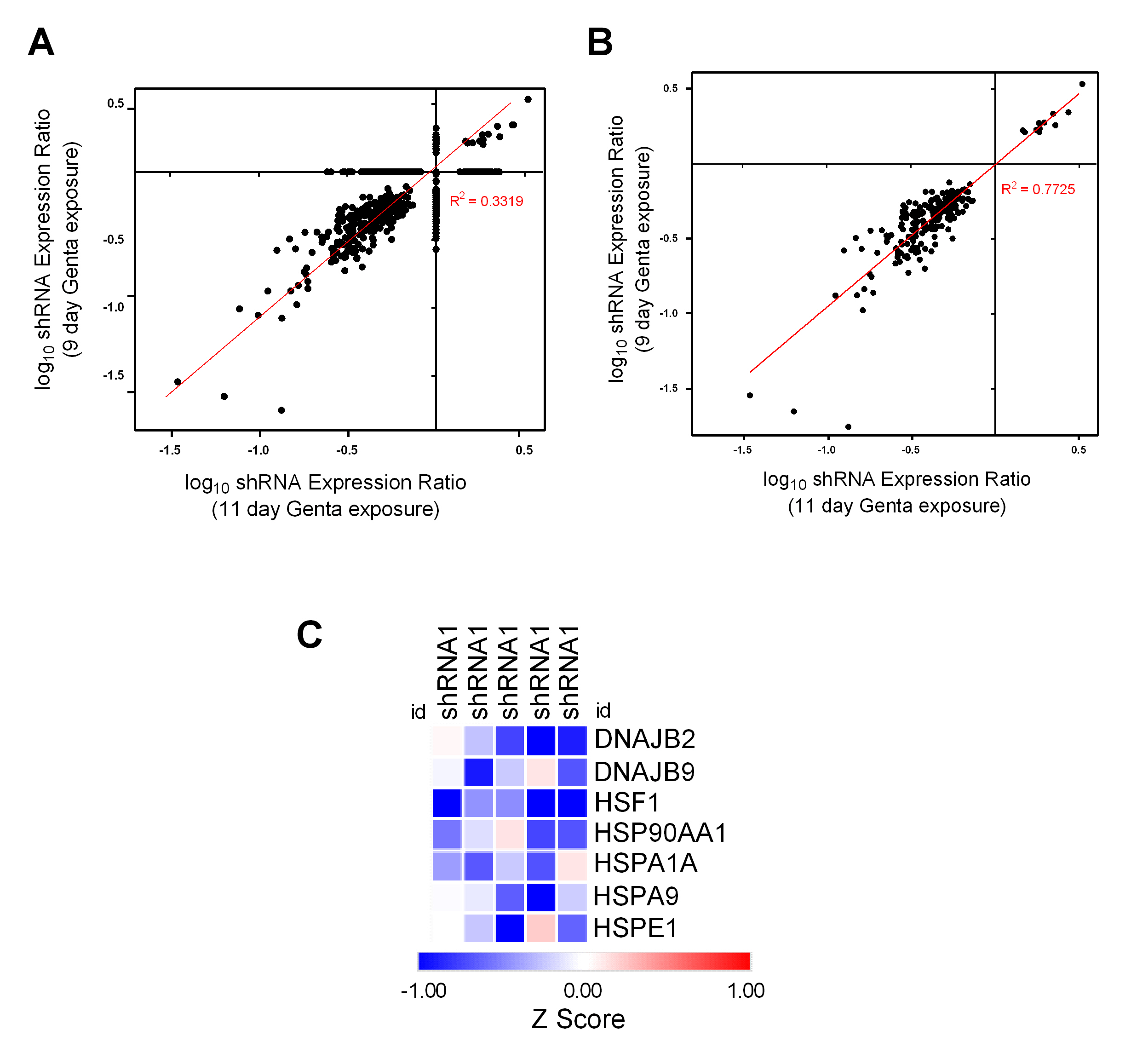

Supplement: Supplementary file 1 — Supplemental Figure 1 [file 41419_2020_2382_MOESM1_ESM.jpg]

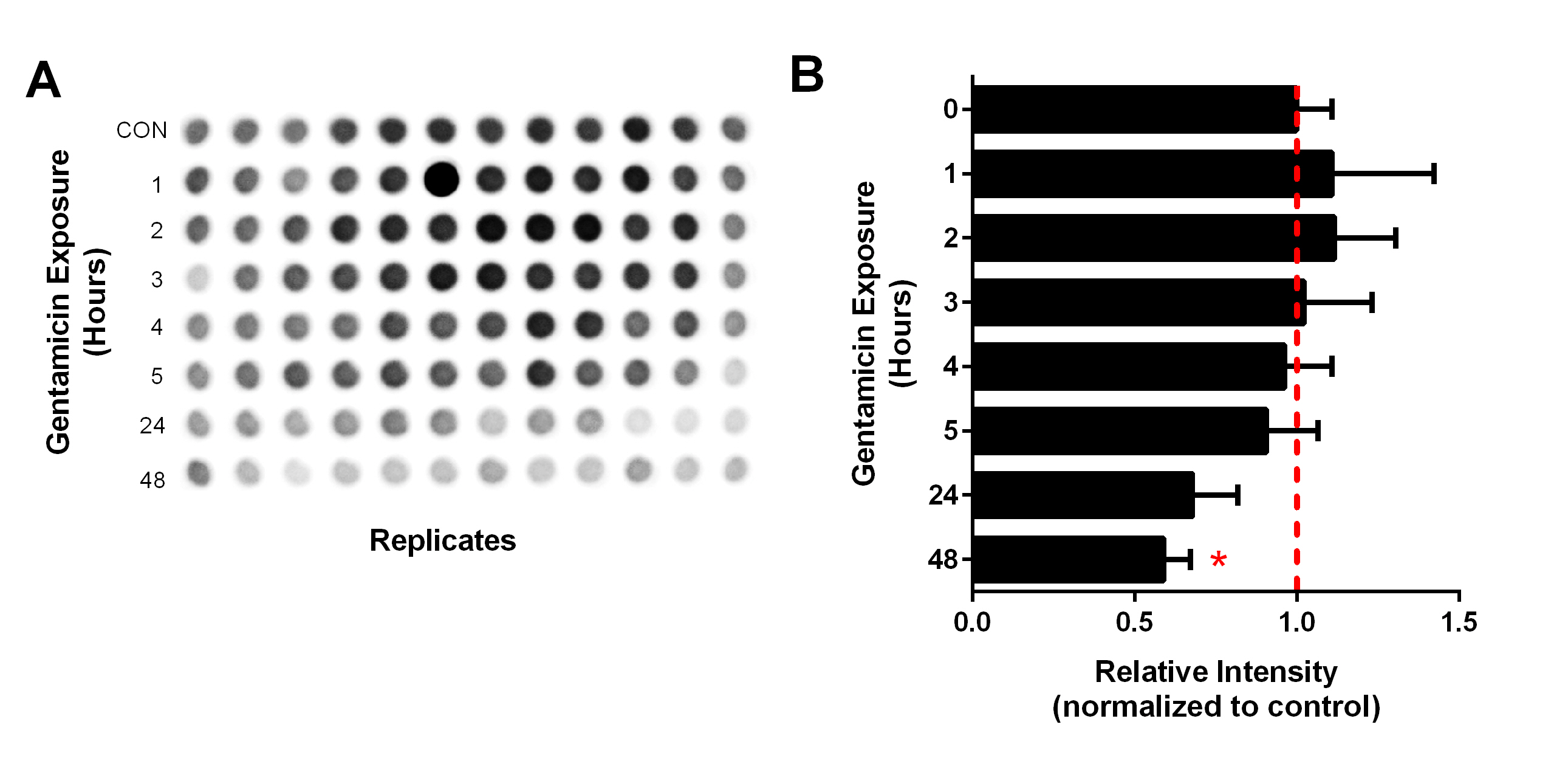

Supplement: Supplementary file 2 — Supplemental Figure 2 [file 41419_2020_2382_MOESM2_ESM.jpg]

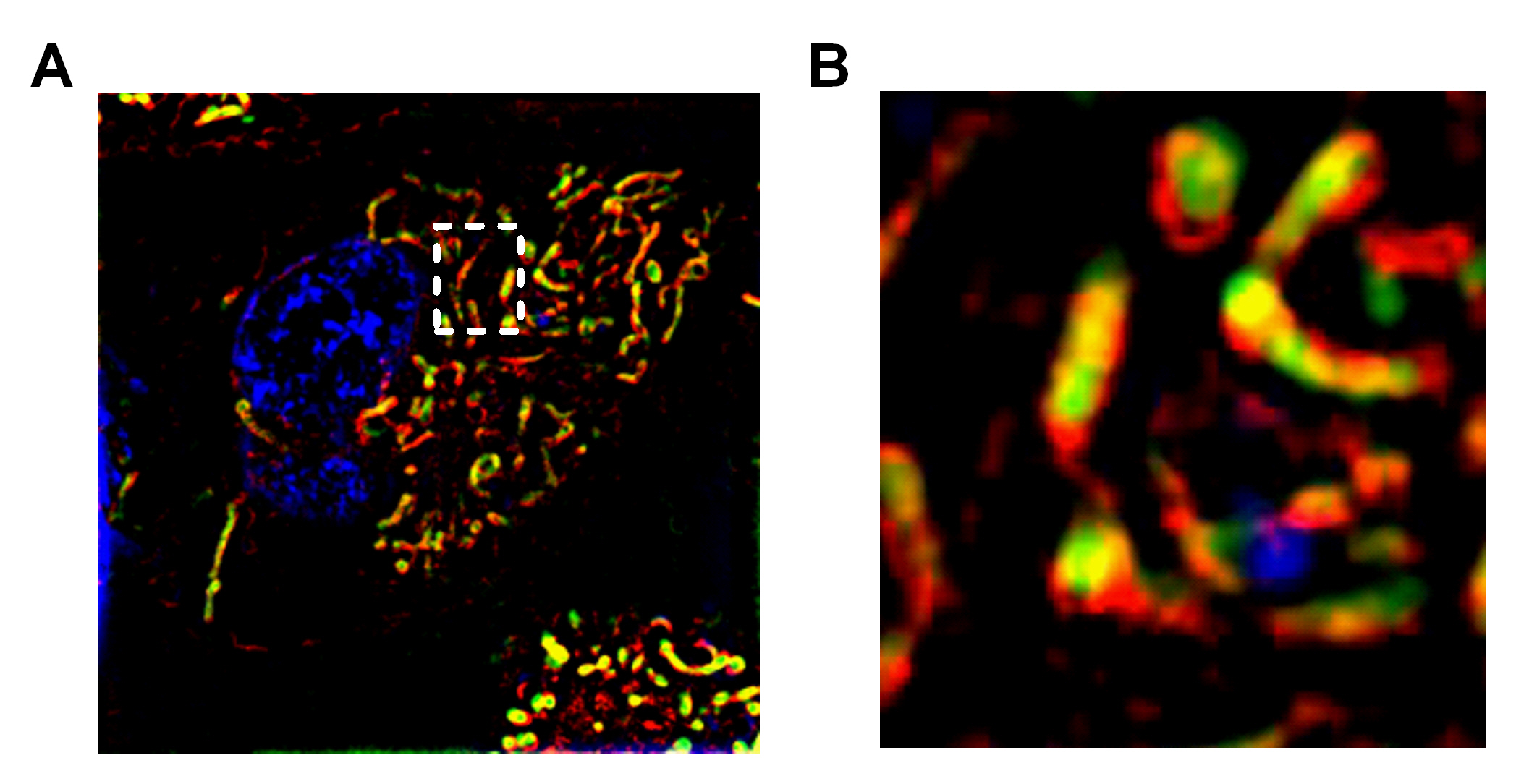

Supplement: Supplementary file 3 — Supplemental Figure 3 [file 41419_2020_2382_MOESM3_ESM.jpg]

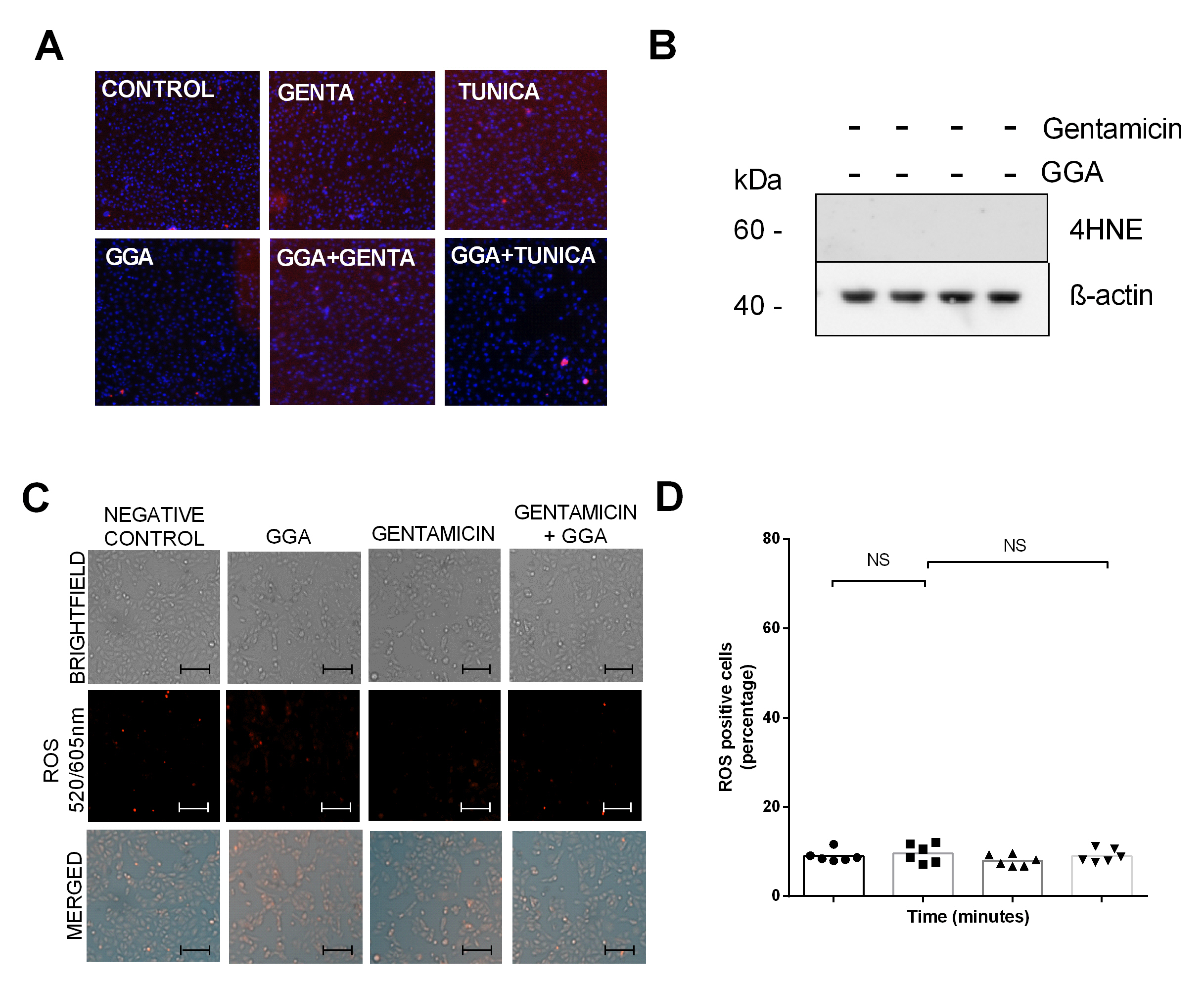

Supplement: Supplementary file 4 — Supplemental Figure 4 [file 41419_2020_2382_MOESM4_ESM.jpg]

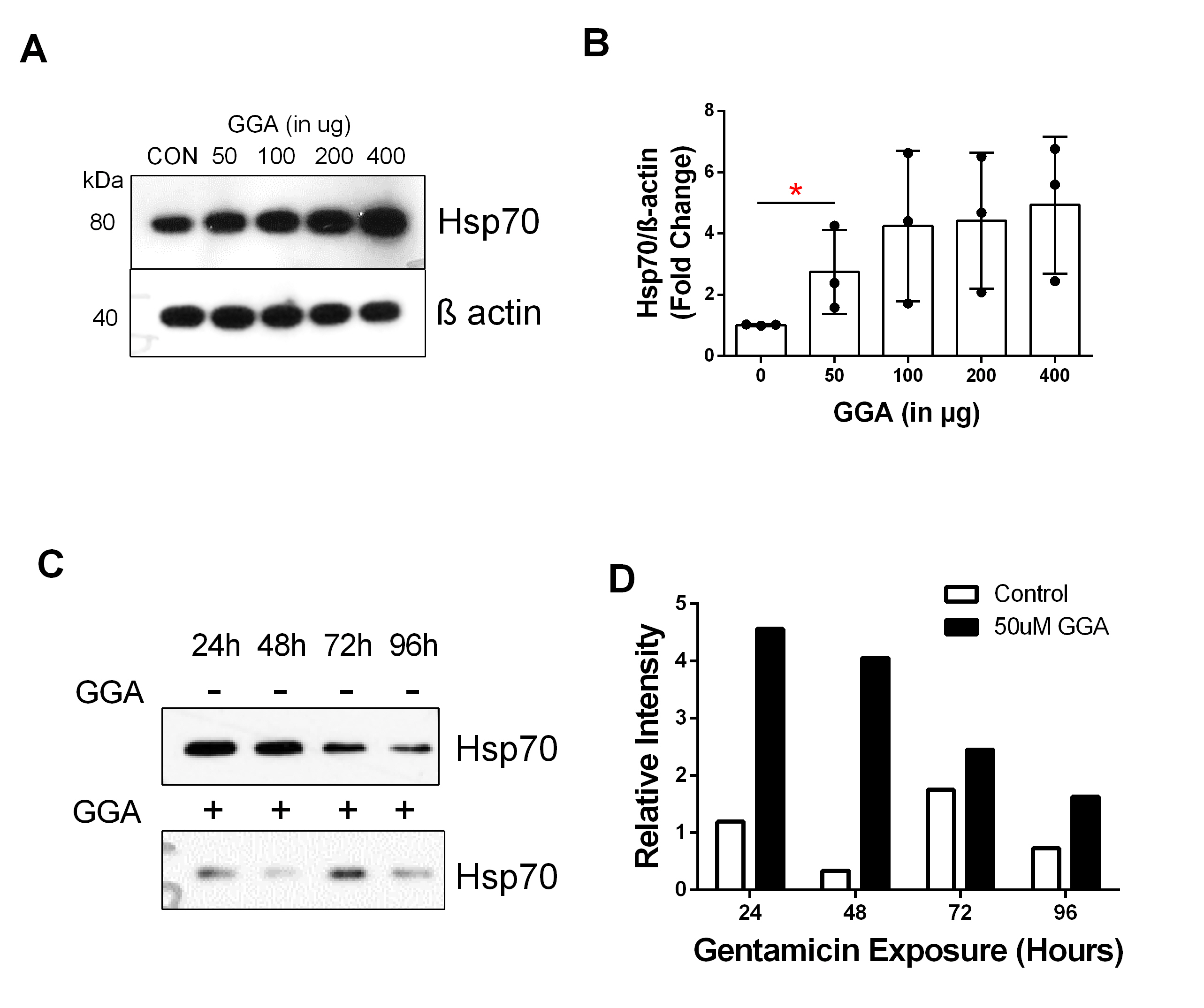

Supplement: Supplementary file 5 — Supplemental Figure 5 [file 41419_2020_2382_MOESM5_ESM.jpg]

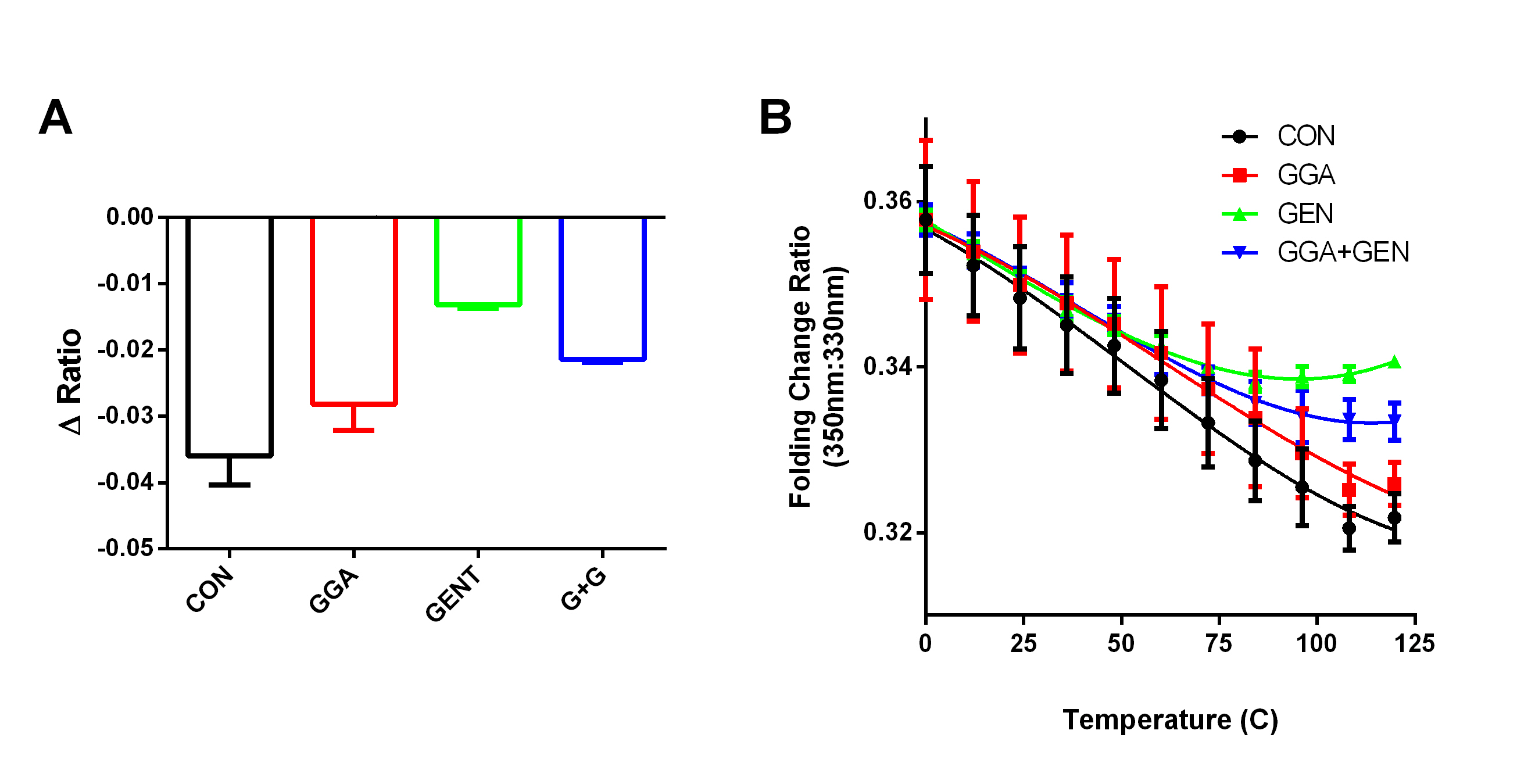

Supplement: Supplementary file 6 — Supplemental Figure 6 [file 41419_2020_2382_MOESM6_ESM.jpg]
