# Supplementary material for: Cross organelle stress response disruption promotes gentamicin-induced proteotoxicity
Source: Cell Death Dis. 2020 Apr 3;11(4):217. doi: 10.1038/s41419-020-2382-7 (PMC7125232; doi:10.1038/s41419-020-2382-7)
Supplement: Supplementary file 7 — Supplemental Table 1 [file 41419_2020_2382_MOESM7_ESM.docx]

| **Gene Abbreviation** | **Name** | **Average shRNA Ratio (Gentamicin:Control)** | **SEM** |
| --- | --- | --- | --- |
| HSF1 | Heat Shock Factor 1 | 0.182 | 0.076377 |
| HSP90AA1 | Heat Shock Protein 90 Alpha Family | 0.541 | 0.209224 |
| HSPA1A | Heat Shock Protein 70 Family | 0.587 | 0.179891 |
| HSPA9 | Heat Shock Protein 70 Family | 0.54 | 0.176829 |
| HSPE1 | Mitochondrial Heat Shock Protein Family | 0.418 | 7.36E-06 |
