# Supplementary material for: Cross organelle stress response disruption promotes gentamicin-induced proteotoxicity
Source: Cell Death Dis. 2020 Apr 3;11(4):217. doi: 10.1038/s41419-020-2382-7 (PMC7125232; doi:10.1038/s41419-020-2382-7)
Supplement: Supplementary file 8 — Supplemental Figures Legends [file 41419_2020_2382_MOESM8_ESM.docx]

**Supplemental Figure 1**: **Duplicate RNAi screens in gentamicin-exposed HK-2 cells showed remarkably similar changes in the abundance of shRNA directed against specific signal pathways.** (**A**) Linear regression of all shRNA abundance changes from duplicate screens showed an R^2^ = 0.3319. (**B**) Linear regression analysis of the 226 signal pathway selected changes in shRNA abundance from duplicate screens showed an R^2^ = 0.7725. (**C**) Heat map of heat shock proteins in these shRNA screens identified a consistent decrease in Hsp70-related shRNA abundance caused by gentamicin exposure. Direct Hsp70 components, such as HSPA1A and HSPA9 were reduced as well as HSF-1, the master regulator of several heat shock proteins; color bar shows relative shRNA abundance.

**Supplemental Figure 2**: **Gentamicin causes progressive loss of luciferase reporter activity.** (**A**) Dot blot analysis of luciferase activity in human renal cells transfected with a luciferase reporter and measured by a standard luciferase assay during 0-48 hr gentamicin exposure. (**B**) Quantitative analysis of luciferase activity normalized to control over 0-48 hr gentamicin exposure; * *P* < 0.05.

**Supplemental Figure 3**: **Multimedia video** **“Gentamicin Exposed Cell Mitochondrial Fragmentation and ER Dissociation Time Lapse”** Uploaded to *Cell Death Disease* Manuscript Submission site. Mitochondria stained with MitoTracker Green, endoplasmic reticulum (ER) stained with ER Tracker Red, and nuclei stained blue with Hoechst dye. At baseline, cells contain elongated mitochondria that closely associate with ER; After 15 minutes of gentamicin exposure, mitochondria fragment but remain co-localized with ER. After 30-minute gentamicin exposure, fragmented mitochondria dissociate from ER; INSETS show magnified views of the areas indicated by white dashed boxes.

**Supplemental Figure 4**: **GGA preserves the CORE prior to changes in whole cell oxidative stress and misfolded protein accumulation**. Compared to vehicle control, brief gentamicin exposure did not alter: (**A**) Thioflavin T staining within 30 min gentamicin exposure; (**B**) 4HNE content within 30 min gentamicin exposure and (**C**) cellular ROS accumulation assessed by fluorescent whole cell oxidative stress assay within 60-minute gentamicin exposure. (**D**) Quantitation of whole cell ROS accumulation after 1 hour of gentamicin exposure; bars = 50 µm; NS “non-significant”; n =6.

**Supplemental Figure 5**: **GGA elicits robust and sustained induction of Hsp70 in renal cells.** (**A,B**) GGA treatment (50-400μM) for 24 hours caused a dose-dependent increase in steady state Hsp70. (**C,D**) Hsp70 induction persists for up to 96 hours after GGA exposure; Error bars = SEM; n=3.

**Supplemental Figure 6**: **Gentamicin decreases temperature-dependent cell protein flexibility in renal cells.** (**A**) Effect of gentamicin *vs*. GGA + gentamicin on cell protein flexibility using a microscale thermophoresis assay. (B) Time course of the effect of gentamicin on of protein flexibility in human renal cell lysates; GGA partially restores protein flexibility in gentamicin exposed cells; n =3.
